# Design and Fabrication of a Ratiometric Planar Optode for Simultaneous Imaging of pH and Oxygen

**DOI:** 10.3390/s17061316

**Published:** 2017-06-07

**Authors:** Zike Jiang, Xinsheng Yu, Yingyan Hao

**Affiliations:** Key Lab of Submarine Geosciences and Prospecting Techniques, Ministry of Education, College of Marine Geosciences, Ocean University of China, Qingdao 266100, China; jiangzike2011@126.com (Z.J.); 15634219925@163.com (Y.H.)

**Keywords:** pH, dissolved oxygen, planar optode, ratiometric, sea-water, rain drops

## Abstract

This paper presents a simple, high resolution imaging approach utilizing ratiometric planar optode for simultaneous measurement of dissolved oxygen (DO) and pH. The planar optode comprises a plastic optical film coated with oxygen indicator Platinum(II) octaethylporphyrin (PtOEP) and reference quantum dots (QDs) embedded in polystyrene (PS), pH indicator 5-Hexadecanoylamino-fluorescein (5-Fluorescein) embedded in Hydromed D4 matrix. The indicator and reference dyes are excited by utilizing an LED (Light Emitting Diode) source with a central wavelength of 405 nm, the emission respectively matches the different channels (red, green, and blue) of a 3CCD camera after eliminating the excitation source by utilizing the color filter. The result shows that there is low cross-sensitivity between the two analytes dissolved oxygen and pH, and it shows good performance in the dynamic response ranges of 0–12 mg/L and a dynamic range of pH 6−8. The optode has been tested with regard to the response times, accuracy, photostability and stability. The applied experiment for detecting pH/Oxygen of sea-water under the influence of the rain drops is demonstrated. It is shown that the planar optode measuring system provides a simple method with low cross-talk for pH/Oxygen imaging in aqueous applications.

## 1. Introduction

For decades, microelectrodes have been widely used in underwater observation, enabling the further understanding of chemical gradients of seawater [1]. However, they can only provide point measurements with high spatial resolution. Recently, a better technique based on the use of an optical oxygen indicator for spatial mapping (called planar optode) has been introduced for real time applications [2,3]. The planar optode based on luminescence of specific indicators allowed multiple measurement of analytes in a variety of formats, and imaging analysis of larger areas. 

So far, the majority of planar optode studies have been focused on pH and oxygen dynamics in rhizospheres [4,5,6]; measurements in sea-ice for resolving physical and biologically induced oxygen dynamics [7]; marine sediments including the investigation of microbial mats and biofilms [8,9]; effects of bioturbating/irrigating fauna [10,11,12]; and studies of pH two-dimensional in benthic substrates [13,14]. There is a broad interest in applying a multi-analyte planar optode to environmental sciences, biological, marine sediments, and medical science benthic communities [15,16,17,18], but the requirements of relatively complicated multi-analyte planar optode, and measuring systems, have limited the number of users. 

The first planar optode systems introduced for aquatic applications were based on pure intensity measurements [19]. Pure intensity measurements for a planar optode suffer from several disadvantages and limitations such as the fluorescence intensity being sensitive to variations in excitation light, background interference, and inhomogeneous indicator [19,20,21]. Holst et al. [22] introduced the superior luminescence life-time-based method to overcome the main disadvantage of the pure intensity measurements. While the lifetime method was superior and overcame the main limitations of the intensity-based systems, it required a relatively complex trigger control circuit and high precision industrial camera [23]. One alternative to the two systems is the ratiometric approach, it can eliminate the deficiencies caused by varied excitation light, background interference, and inhomogeneous indicator, while not requiring complex available hardware [24,25,26]. 

The previous studies of the ratiometric sensors are mostly based on the DSLR (Digital Single Lens Reflex) or CCD (Charge Coupled Device) cameras with single CCD. The majority of digital cameras make use of the bayer color filter, which allows them to separate the incident light into the three primary colors: red, green, and blue. However, it detects only one-third of the color information for each pixel. The other two-thirds must be interpolated with a demosaicing algorithm to 'fill in the gaps', resulting in a much lower effective resolution [27]. In this scheme, it could cause two-thirds of light intensity loss, and affect the sensitivity of the planar optode. In this paper we present an imaging observation system based on 3CCD camera. The imaging system of 3CCD camera uses three separate charge-coupled devices (CCDs), each one taking a separate measurement of the primary colors, red, green, or blue light. The incident light coming into the lens is split by a trichroic prism assembly, which directs the appropriate wavelength ranges of light to their respective CCDs. Compared to cameras with single CCD, 3CCD cameras generally provide superior image quality through enhanced resolution and lower noise. By taking separate readings of red, green, and blue values for each pixel, 3CCD cameras achieve much better precision than single-CCD cameras, and ensure the spectral intensity.

In order to simultaneously realize the imaging of pH and oxygen chemical parameters, multiple indicators can be combined within a single planar optode [28,29]. It is very difficult to separate the emission of the dyes, and this would cause cross-sensitivity between the red, green and blue channel of a 3CCD camera. Moßhammer et al. introduced a new optical dual-analyte sensor for imaging. It is a simple way to use a 2CCD camera with near-infrared (NIR) sensitive camera chip to expand the potential of ratiometric readout [30]. 

The 3CCD camera can be used to read out one reference dye and two indicator dyes. However, the principle is limited by the spectral overlap of the traditional dyes possessing broad emission spectrum. This calls for simpler, lower overlap, good antijamming capability and user-friendly dyes and measuring setups. Owing to the characteristics of insensitivity, broad spectrum excitation, tunable emission wavelength, narrow half peak width, good light stability and high quantum yield [31], quantum dots (QDs) is an excellent reference. Wang et al. introduced a sensing film doped with modified water-soluble CdTe/CdS quantum dots and PtF20-TPP imbed in sol-gel with two layers respectively on slides. The thickness of the sensing film is about 70 µm, and it achieved colorimetric oxygen determination with precise, distinct, and tunable color [32]. In order to improve homogeneity, mechanical strength and applicability of the sol-gel in extreme condition, we synthesized the oleic acid modified QDs which was lipophilic and could be imbed in polystyrene (PS) layer without leakage in aquatic conditions.

In this study, we synthesized the oleic acid modified QDs [33,34] with narrow spectral emission. It was lipophilic, high-efficiency and pH/oxygen insensitive. By doping the QDs as the reference dye, a new ratiometric pH/Oxygen planar optode combination with a 3CCD camera was designed for simultaneous imaging of pH and oxygen. The planar optode used PtOEP as the oxygen indicator with red emission, 5-Fluorescein as the pH indicator with green emission, and QDs as the reference dye with blue emission. These three dyes were respectively embedded in polystyrene (PS) and Hydromed D4 matrix. The performance of the planar optode was validated for detecting pH/ Oxygen of sea-water under the influence of the rain drops [35,36]. 

## 2. Experimental

### 2.1. Materials Preparation

The oxygen indicator dye PtOEP (CAS number : 31248-39-2), pH indicator dye 5-Fluorescein (CAS number: 73024-80-3), chloroform, ethanol, octadecene (ODE), TOP (trioctylphosphine) and oleic acid (OA) were all analytical grade, they were purchased from Aladdin Chemical Company (Shanghai, China); The matrix PS, D4 were purchased from J&K Chemical Company (Shanghai, China). 

### 2.2. Synthesis of Lipophilic QDs

The oleic acid modified QDs was synthesized according to the literature [37]. Briefly, The 70 mL clear octadecene (ODE) solution was prepared by mixing t 5.54 g OA, 0.51 g CdO and was heated at 180 °C under N_2_ atmosphere. We could obtain different sizes of QDs by controlling the temperature respectively, and herein the mixture was heated to 275 °C. Fully stirred TOP-Se solution containing 1.3 mmol Se powder, 0.5 g trioctylphosphine and 10 mL ODE, was quickly injected into the three-neck round-bottom flask. Finally, the resulting stable QDs solution was dispersed in chloroform, and stored at a temperature of 8 °C. 

### 2.3. pH/Oxygen Planar Optode Fabrication

**Figure 1:**
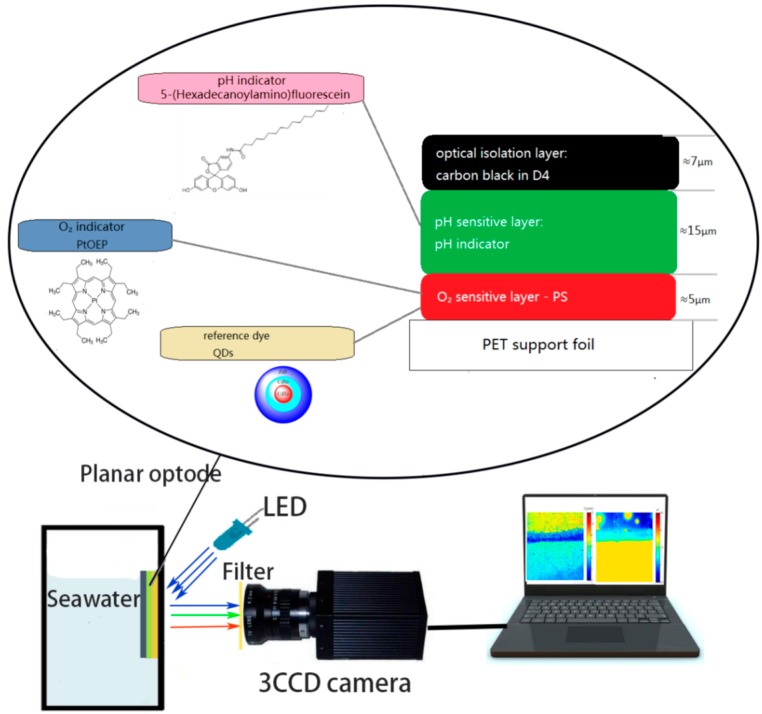
Schematic drawing of the new ratiometric multiple pH/Oxygen planar optode (the oval part) and measuring system with JAI AT-200GE 3CCD camera. The oval part indicates the drawing of the pH/Oxygen sensor composed of a three layer system containing the depicted indictor and reference dyes. Filter: 455 nm longpass. LED: high power LED (peak wavelength 405 nm) as a light source.

For the oxygen layer, the solution was fabricated by mixing 200 mg of polymer PS, 1 mg of QDs, 1 mg of PtOEP dye in 0.4 mL chloroform. The sensor film was fabricated by utilizing a knife coating which was a fast and simple method [3]. The solution was coated onto a 125 µm thick polyester support foil to form the a ~5 μm thick oxygen sensitive layer after solvent evaporation. The solution containing 200 mg of polymer D4 (ethanol: water, 9:1 w/w), 1 mg of 5-Fluorescein dye in chloroform was knife coated on top of the oxygen layer, obtaining ~15 μm thick pH sensitive layer. For the optical isolation layer, the solution containing 1.00% w/w of carbon black in 10% w/w of Hydromed D4 (ethanol: water, 9:1 w/w) was coated onto the pH layer after solvent evaporation (Figure 1). 

The ratiometric measuring scheme was sensitive to wavelength-dependent scattering and reflection from any background behind the transparent sensor. However, a thin translucent D4 layer imbedded with carbon powder solved the problem. Thus, the pH/Oxygen sensitive film was coated with D4 matrix doped with carbon powder. The dry isolation layer was ~7 µm thick and semitransparent with a light transmission of ~20%. This ensured that any structures behind the sensor were still visible during oxygen measurements, without affecting the ratiometric approach.

### 2.4. Instrument and Method

The pH/Oxygen measuring system is illustrated in Figure 1. For this present work, the luminescence excitation was provided by 6 high power 405 nm LED (λ-peak = 405 nm, Φ = 400 mW at I_F_ = 1100 mA) from Tianyao companies, and the LED was powered by a DH171 5A-3 DC stabilized power supply (Dahua Company, Beijing, China). The relative luminescence intensity was measured using the Ocean Optics spectrometer USB2000+ and JAI AT-200GE 3CCD camera (Daheng Image Company, Beijing, China). Emissions filters which were fabricated in front of the 3CCD camera were 440 nm long pass filters (Nantong Optical glass Company, Beijing, China).

The JAI AT-200GE camera provided 2-megapixel resolution (1628 × 1236) based on three 1/1.8-inch CCDs, 1624 (h) × 1236 (v) effective pixels (4.40 µm square) for each CCD. With 3-CCD technology, a specific R, G, and B value was captured for each pixel. The camera has a GigE Vision interface and its output can be either 24-bit or 32-bit RGB in TIFF format. The 32-bit RGB TIFF images were automatically saved onto the computer (exposure time of 15535 μs) and used for further calculations. Processing of the recorded images was performed with the free software ImageJ (http://rsbweb.nih.gov/ij/) and analyzed in MATLAB software. 

**Figure 2:**
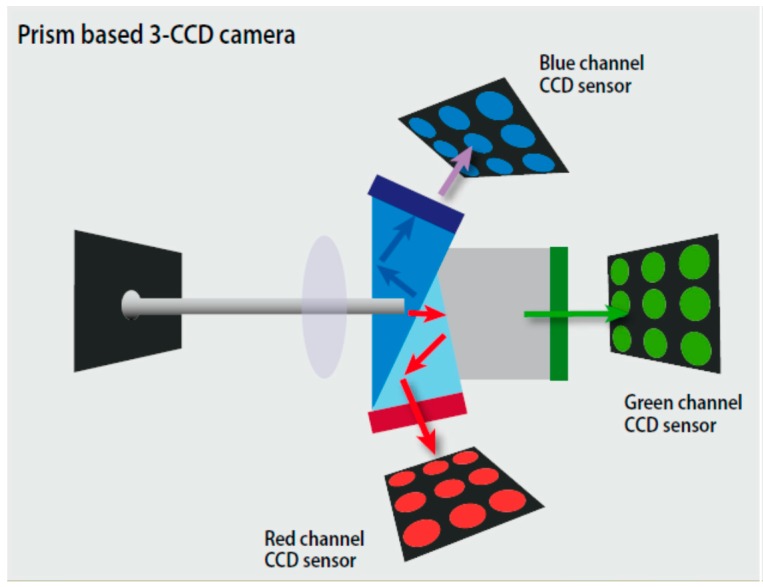
Schematic drawing of prism based JAI AT-200GE 3CCD camera.

Bayer mosaic color cameras used a pattern of color filters and an interpolation process to estimate the approximate RGB value of a given pixel. With 3-CCD technology, a specific R, G, and B value was captured for each pixel (Figure 2). This inherently produced higher color precision in the 3-CCD output. Furthermore, the spectral curves resulting from the hard dichroic prism coatings were much steeper than the curves from the soft polymer dyes used in Bayer filters. This enabled the 3-CCD cameras to produce exceptionally accurate color data without the uncertainty that comes from the overlap regions. In addition to reducing color precision, the overlap in the color filter response also causes part of each pixel’s well capacity to fill with photons resulting from the crosstalk, thus decreasing the available well capacity. Precision response from the dichroic coatings enables each channel to efficiently use the full well capacity of the pixel, allowing the maximum possible dynamic range. 

The oxygen concentrations in 30‰ salinity seawater was controlled by mixing oxygen and nitrogen, and this process was controlled by gas flow-meters. The pH calibration solution was confected by the 30‰ artificial seawater containing 10 mM TRIS-HCL buffer. The pH of the seawater was adjusted by adding either 1 M HCl or 1 M NaOH to the glass tank. The dissolved oxygen concentration was measured by O_2_ microelectrode (Unisense O_2_ Microsensor, Aarhus, Denmark), and the temperature-compensated pH microelectrode (YSI pH 100A, Shanghai, China) was used to continuously monitor the pH level of the seawater. 

### 2.5. Simulated Rainfall Experiments

For sea-water measurements, the pH/Oxygen sensitive film was transferred to a custom-made quartz aquarium (H × L × W: 12 × 12 × 12 cm) that was subsequently filled with natural seawater collected in the Shilaoren Bay, Qingdao, China. The quartz aquarium was allowed to settle for 24 h before the presented images were recorded. During this period, the setup was kept at a constant temperature of 17 °C and exposed to a 12/12 h light/dark cycle. We simulated rainfall at the rate of about 0.5 inch/h for 10 min by experimental rainfall setup in a laboratory. A device with an array of micro holes of constant sizes (3 mm in diameter) was used to produce rain of constant size drops. The rainwater (the oxygen concentrations of 8.31 mg/L, pH value 7.2) was collected and stored in the tank with an air pump, and the air pump is a device for pushing air during this period.

## 3. Results and Discussion

### 3.1. Optical Properties of Multiple pH/Oxygen Planar Optode

**Figure 3:**
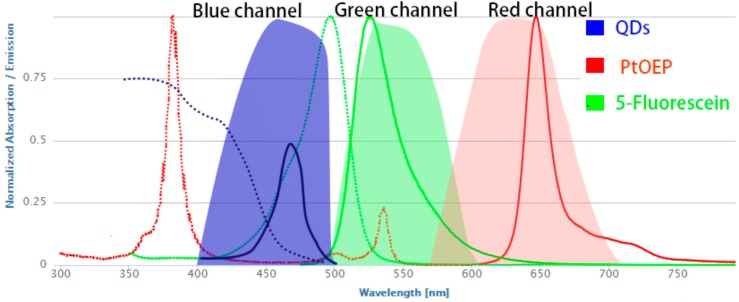
Spectra of the dyes (absorption in dashed and emission in solid lines), and the spectral range of three different channels (red, green, and blue) in JAI AT-200GE 3CCD camera.

Figure 3 showed the luminescence spectra of three dyes materials (PtOEP, 5-Fluorescein, and QDs) at room-temperature. When excited with a 405 nm LED, the planar optode exhibited intense luminescence emissions at 650 nm, 520 nm, and 460 nm, respectively. The emissions of QDs served as the reference insensitive to pH and oxygen, and it also acted as an internal donor molecule for the indicator. Since the emissions of the QDs overlapped with the absorption of 5-Fluorescein, the brightness of the two indicators, especially the 5-Fluorescein was enhanced by making use of QDs. The emissions of indicator PtOEP and 5-Fluorescein are respectively sensitive to oxygen and pH, thus the pH and oxygen can be measured. In addition, by the ratio process, the deficiencies of the intensity was eliminated by the reference QDs. 

The emission intensity of the three dye materials (PtOEP, 5-Fluorescein, and QDs) respectively was recorded by the corresponding three spectrum channels (red, green, and blue) of the 3CCD camera. The pixel intensity of the red, green channel was dominated by the luminescence from the PtOEP and the 5-Fluorescein, respectively. The blue channel represented the luminescence of QDs and blue LED excited light, after eliminating the excitation source by utilizing the longpass filter (>455 nm). There was a negligible spectral overlap between the three dye materials. 

### 3.2. Calibration and Accuracy Evaluation of the pH/Oxygen Planar Optode

**Figure 4:**
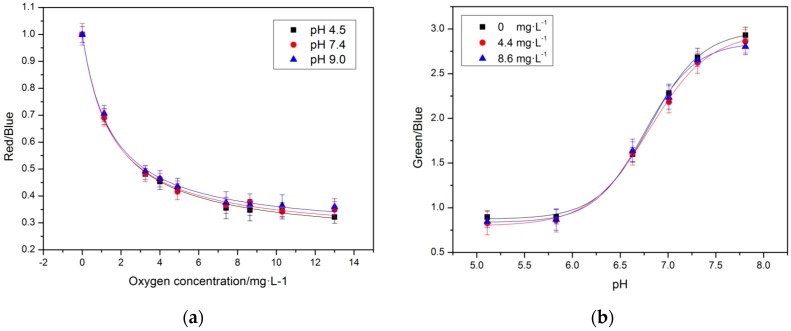
The calibration curves for (**a**) oxygen and pH (**b**) in seawater. (17.0 ± 0.2 °C).

Figure 4 showed the fit equations and calibration plots for pH and oxygen in artificial sea water at a concentration of 30‰ (salinity). For the oxygen calibration, the relationship between intensity and oxygen concentration was reflected by the modified Stern–Volmer equation [38]:

(1) $$\frac{I}{I_{0}}=\left(A+\frac{1-A}{1+K_{\mathrm{sv}}\left[O_{2}\right]}\right)$$

where, I was the intensity ratios of the red and blue channels (R/B) of the planar optode at different oxygen concentration, I_0_ expressed the corresponding values at oxygen free condition. Ksv and A respectively were the constant of calibration curve and the quenching ratio of indicator. In an ideal quencher system, there is a linear relationship between I_0_/I and oxygen concentration, so a linear Stern-Volmer equation can be applied. However, a matrix effected on the quenching properties of luminescent dyes in solid solutions strongly depends on the polymer used. It is necessary to take into account the influence of polymer which was attributed to a distribution of quenching rate constants. 

For the pH calibration, the relationship between intensity and pH values was fitted by the four-parametric Boltzmann (sigmoidal) equation [39]:

(2) $$R=b+\frac{a-b}{1+e^{\frac{\mathrm{pH}-\mathrm{pka}}{\mathrm{dx}}}}$$

where, R was the intensity ratios of the green and blue channels (G/B) of the planar optode at different pH values, a, b and dx respectively express the empirical parameters and the width of the curve, pKa was the coefficient of center of the calibration curve. 

**Figure 5:**
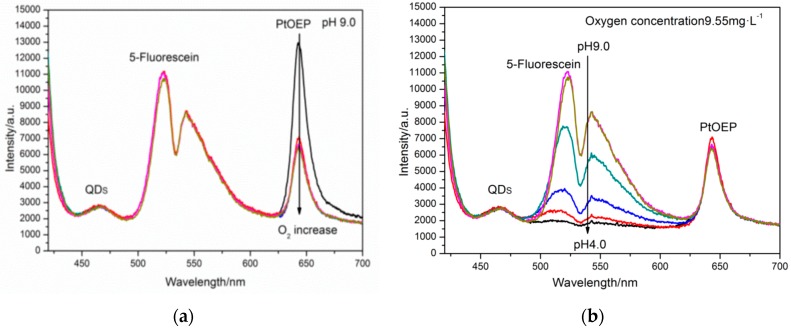
The emission spectra of ratiometric pH/oxygen planar optode:(**a**) Emission spectra in different oxygen concentration at pH 9.0, the different colors of the lines represented the different oxygen concentration, and the arrow indicates the increasing of oxygen concentration; (**b**) Emission spectra at pH values between 4 and 9 under the oxygen concentration of about 9.55 mg·L^−1^, the different colors of the lines represented the different pH value, and the arrow indicated the decrease of pH.

In order to evaluate potential cross-talk for oxygen and pH, the experiments were conducted at varying condition (Figure 5). Figure 4a presented the calibration curves for the oxygen sensitivity in the dual pH/Oxygen planar optode system, and these were conducted for three different pH values: one acidic (pH 4.5), alkalescence (pH 7.4) and one alkaline (pH 9.0).

The optode’s response for the oxygen showed nonlinear calibration curves with maximum sensitivity at low oxygen concentrations. The ratio decreases more than 80% when the oxygen concentration increases from 0 to 8 mg/L. For this oxygen concentration scope, the pH/Oxygen planar optode retained a highly effective large reduction. This was mainly because of the simultaneous presence of static and dynamic quenching [40] and the inequality of the microenvironment of the immobilized dye PtOEP placed within the polymer matrix PS. In the oxygen concentration of 0–6 mg/L, the calibration curves exhibited no cross interferences caused by changes in the pH. However, a slight cross-sensitivity toward oxygen was observed in the oxygen concentration of 8–12 mg/L. This can be explained by the energy transfer to the PtOEP from the luminescence of 5-Fluorescein, the interference increased with decreased luminescence of PtOEP under hyperoxia condition. The pH response of the dual pH/Oxygen planar optode followed a sigmoidal pattern with an apparent pKa of around 6.95. As the Figure 4b showed, a minor cross-sensitivity toward oxygen was observed at pH values around the pKa. In the pH scope between 6.3 and 7.3, the calibration curves exhibited no cross interferences caused by changes in the oxygen.

Table 1 and Table 2 illustrated the accuracy evaluation between the pH/Oxygen planar optode and pH/oxygen microelectrode. It was noted that the pH/Oxygen planar optode possessed superior behavior in oxygen depleted condition for the concentration range of 0–6 mg/L than the supersaturated condition. For the pH values, the optode with an apparent pKa of around 7.15 showed superior behavior at pH values between 6 and 8. 

### 3.3. Long Term Stability and Photostability

**Figure 6:**
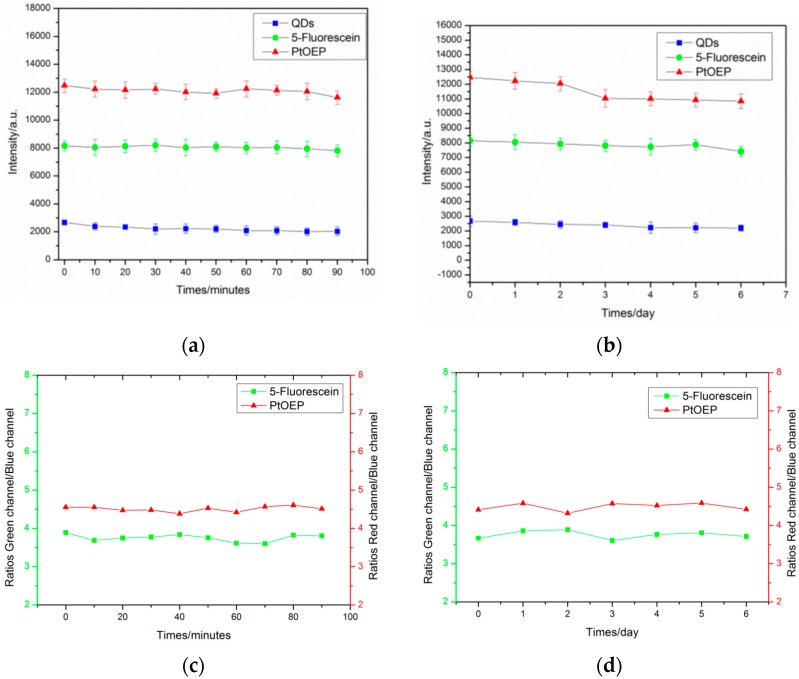
Photostability and long term stability of the pH/Oxygen planar optode. (**a**,**b**) The relative luminescence intensities of the QDs, 5-Fluorescein, and PtOEP (Platinum(II) octaethylporphyrin). (**c**,**d**) The ration of the pH/Oxygen planar optode: ratios of Green channel/Blue channel, and ratios of Red channel/Blue channel.

The stability and photostability was a key analytical figure of the pH/Oxygen planar optode [26,41]. The photostability was tested by placing the ratiometric pH/Oxygen planar optode into seawater with continuous irradiation by using a USHIO EKE, 150W halogen lamp (Weiguang Company, Shenzhen, China) equipped with an optical filter at room temperature for around 100 minutes. The LI-250A photometer (ECOTEK Company ,Beijing, China)was used to measure the output of halogen lamp, and the irradiances was 253 μmol photons·s^−1^·m^−2^. The relative luminescence intensities of the reference QDs, 5-Fluorescein, and PtOEP decreased 9.82%, 4.701%, 6.45%, respectively (Figure 6a). The ration of the Green channel/Blue channel changed 4.67%, and ratios of Red channel/Blue channel changed 5.25% (Figure 6c).

For the stability test, the experiment was conducted to evaluate the pH/Oxygen planar optode by putting the planar optode in artificial sea-water (salinity 30‰) at 25 °C for a period of 1 week. Figure 6b showed that there was no obvious leakage for the period time, the relative luminescence intensities of the reference QDs, 5-Fluorescein, and PtOEP decreased 12.15%, 9.01%, 13.55%, respectively (Figure 6b). The ration of the Green channel/Blue channel changed 7.43%, and ratios of Red channel/Blue channel changed 8.38% (Figure 6d). The photostability of the pH/Oxygen planar optode was stable. For the stability and photostability, the ratio is less unaffected and more superior than the intensity. In the process of continuous long-term use, in order to assure the accuracy of the measurement result, the pH/Oxygen planar optode needed regular calibration.

In order to evaluate the possibility of migration of the lipophilic fluorescein into the optical isolation layer, and into the seawater, experiments were conducted. We analyzed the fluorescence of seawater used in the quartz aquarium during the stability experiment. The negligible fluorescence was detected when the seawater was excited by a 405 nm LED, so the effect of migration was negligible.

### 3.4. Response Time of the pH/Oxygen Planar Optode

In real-time applications measurements, response time (time to reach 90% of the full signal) was a critical performance factor. The response times of the pH/Oxygen planar optode (from 9.55 mg/L to 0 mg/L) are around ~35 s, ~2 s (from 0 mg/L to 9.55 mg/L), and ~16 s for pH changes (from 5 to 8). The signal changes of the pH/Oxygen planar optode were fully reversible.

## 4. Application of the Planar Optode for Imaging oxygen and pH Simultaneously within Seawater

**Figure 7:**
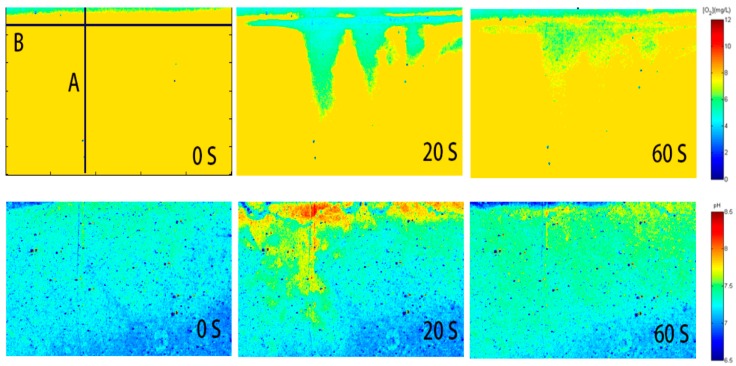
Time series recording of the pH/oxygen distribution. The Line A represented extracted vertical profiles of the raindrops landing area, the Line B indicated the position of air-water interface.

In order to test the applicability of the pH/Oxygen planar optode on the sample, the seawater was collected (in Shilaoren Bay, Qingdao, China) and analyzed. By applying the pH/oxygen planar optode, the results in Figure 7 showed the measurement of the two-dimensional pH/oxygen distribution of sea-water under the influence of the rain drops. The scale of pH and oxygen concentration was respectively expressed with color bars. 

**Figure 8:**
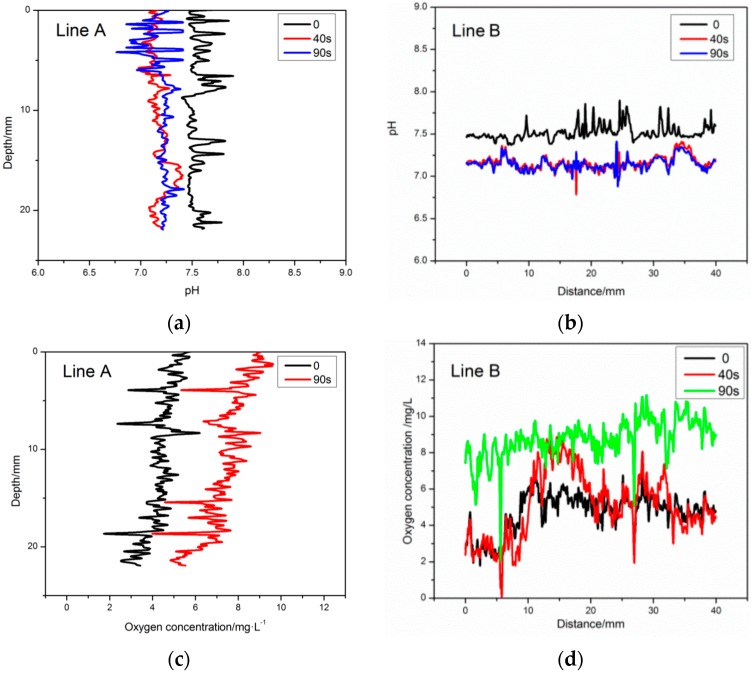
Extraction of time-resolved and depth-resolved dynamics from time series recording pH/oxygen distribution images in Figure 7. (**a**,**c**) The vertical and horizontal pH/oxygen profiles of Line A in Figure 7; (**b**,**d**) The horizontal pH/oxygen profiles of Line B in Figure 7.

To analyze the pH and oxygen dynamics of the sea-water under the influence of the rain drops in more detail, the vertical and horizontal profiles (corresponds to the marked line A, B in Figure 8) of the pH/Oxygen were extracted from the two-dimensional pH/oxygen figure. The results showed that the rainfall could cause significant changes of dissolved oxygen and pH value of the water surface in a vertical and horizontal direction. On the surface of the water to a vertical depth of 23 mm, the change of dissolved oxygen was the most obvious: the content of oxygen increased 2.3 mg/L within 40 seconds after rainfall, the pH value decreased to 7.2. To a vertical depth of 12 mm, the diffusion of dissolved oxygen and pH value was slow. It is proved that the change of dissolved oxygen content and pH value in the process of regulating the surface water was affected by the rain drops in the surface water in the region with a small variation of wind speed, temperature or pressure. This study provided a new technical method for understanding the influence of raindrops on the dissolved oxygen concentration and pH of the surface water in low wind impact areas or static water areas.

## 5. Conclusions

In this work, a novel dual ratiometric pH/Oxygen planar optode combination with a 3CCD camera for simultaneous imaging of pH and oxygen was developed and applied on sea-water under the influence of the rain drops. The planar optode used PtOEP as the oxygen sensitive dye with red emission, 5-Fluorescein as the pH sensitive dye with green emission, and QDs as the pH/Oxygen insensitive reference dye with blue emission. These three dyes were respectively embedded in polystyrene (PS) and Hydromed D4 matrix. The reference QDs and indicator dye was chosen due to its superior optical property, stability, and high lipophilicity. The results indicated that the pH/Oxygen planar optode can be an effective simple, high resolution approach for simultaneous measurement of dissolved oxygen (DO) and pH. This study provides a new technical method for the pH/Oxygen planar optode. It could simultaneously visualize the dynamic changes in pH and oxygen, and have a good nolinear response within dissolved oxygen concentration between 0 and up to 12 mg/L and a pH range from 7 to 9. These specific properties made the pH/Oxygen planar optode suitable for applications in the environmental monitoring field. 

## Figures and Tables

**Table 1:** Comparison of the pH/Oxygen planar optode and electrodes for oxygen measurements. AE: absolute error; RE: relative error; SD: standard deviation.

Oxygen Electrodes	pH/Oxygen Planar Optode			
Measured	Calculated	AE	RE	SD
0.01	0.0096	0.0004	4.00%	0.0038
4	3.884	0.116	2.90%	0.1244
7	7.432	0.432	6.17%	0.3102
9.34	9.974	0.634	6.79%	0.7352
10.59	9.812	0.778	7.35%	0.6321

**Table 2:** Comparison of the pH/Oxygen planar optode and electrodes for pH measurements. AE: absolute error; RE: relative error; SD: standard deviation.

pH Electrodes	pH/Oxygen Planar Optode			
Measured	Calculated	AE	RE	SD
4.23	3.92	0.31	7.32%	0.0462
4.89	4.63	0.26	5.32%	0.1487
5.56	5.80	0.24	3.72%	0.1292
6.45	6.27	0.18	2.79%	0.3043
7.75	7.45	0.3	3.87%	0.2945

## Abbreviations

The following abbreviations are used in this manuscript:

DO	dissolved oxygen
PtOEP	Platinum(II) octaethylporphyrin
PS	polystyrene
5-Fluorescein	5-Hexadecanoylamino-fluorescein
D4	Hydromed D4
TOP	trioctylphosphine
OA	oleic acid
DC	direct current
ODE	octadecene
QDs	Quantumdots

## Appendix A. Matlab Script to Generate Colormap Images of Spatial Oxygen and pH

Im=imread('E:13.tif');
Imcut=imcrop(Im,[650,650,499,499]);
Imd=double(Imcut);
 
Imdr=Imd(:,:,1);
Imdg=Imd(:,:,2);
Imdb=Imd(:,:,3);
  h=ones(3,3);
  h(1,1) = 0;
     h(1,3) = 0; 
    h(3,1) = 0;
  h(1,3) =0;
Imdr = medfilt2(Imdr);
  Imdg = medfilt2(Imdg);
  Imdb = medfilt2(Imdb);
Imd2=zeros(m5,n5);
for i=1:m5
        for j=1:n5
           temp1=Imdr(i,j);
           temp2=Imdb(i,j);
           aver1=mean(temp1(:));
           aver2=mean(temp2(:));
           Imd2(i,j)=aver1/aver2;         end
end
Idmd2=Imd2./I0;
for i=1:m5
    for j=1:n5
        if Imd2(i,j)>1
            Imd2(i,j)=1;
        end
    end
end
do2=((1-A)./(Idmd2-A)-1)./K;
for i=1:m5
   for j=1:n5
       if do2(i,j)>14
           do2(i,j)=14;
       end
       if do2(i,j)<=0
           do2(i,j)=0;
       end
   end
end
imagesc(do2)
h=colorbar;
set(get(h,'Title'),'string','[O_2](mg/L)');
axis off
 
clear; clc;
Im=imread('E:12.tif');
Imcut=imcrop(Im,[590,420,500,500]);
Imd=double(Imcut);
 m5=500
 n5=500
Imdr=Imd(:,:,1);
Imdg=Imd(:,:,2);
Imdb=Imd(:,:,3);
 
  h=ones(3,3);
  h(1,1) = 0;
    h(1,3) = 0;
   h(3,1) = 0;
  h(1,3) =0;
Imdr = medfilt2(Imdr);
 Imdg = medfilt2(Imdg);
 Imdb = medfilt2(Imdb);
 
Imd2=zeros(m5,n5);
for i=1:m5
        for j=1:n5
            temp1=Imdg(i,j);
            temp2=Imdb(i,j);
            aver1=mean(temp1(:));
            aver2=mean(temp2(:));
            Imd2(i,j)=temp1./temp2;
        end
end
for i=1:m5
    for j=1:n5
        if Imd2(i,j)<0
           Imd2(i,j)=0;
        end
    end
end
pH=7.13719+0.2310*log(-1.47284./(Imd2-2.67781)-1);
pH=real(pH)
imagesc(pH)
h=colorbar;
set(get(h,'Title'),'string','pH');
axis off
